# Assessing Quality of Life with the Novel QLQ-CAX24 Questionnaire and Body Composition Parameters in Rectal Cancer Patients: A Single-Center Prospective Study

**DOI:** 10.3390/nu16244277

**Published:** 2024-12-11

**Authors:** Marco Cintoni, Marta Palombaro, Pauline Raoul, Giuditta Chiloiro, Angela Romano, Elisa Meldolesi, Flavia De Giacomo, Elena Leonardi, Gabriele Egidi, Futura Grassi, Gabriele Pulcini, Emanuele Rinninella, Esmeralda Capristo, Antonio Gasbarrini, Maria Antonietta Gambacorta, Maria Cristina Mele

**Affiliations:** 1 UOC di Nutrizione Clinica, Dipartimento di Scienze Mediche e Chirurgiche, Fondazione Policlinico Universitario A. Gemelli IRCCS, Largo A. Gemelli 8, 00168 Rome, Italy; marco.cintoni@unicatt.it (M.C.); marta.palombaro@guest.policlinicogemelli.it (M.P.); elena.leonardi@guest.policlinicogemelli.it (E.L.); gabriele.egidi@policlinicogemelli.it (G.E.); futura.grassi@policlinicogemelli.it (F.G.); gabriele.pulcini@guest.policlinicogemelli.it (G.P.); emanuele.rinninella@unicatt.it (E.R.); mariacristina.mele@unicatt.it (M.C.M.); 2 Centro di Ricerca e Formazione in Nutrizione Umana, Università Cattolica del Sacro Cuore, 00168 Rome, Italy; esmeralda.capristo@unicatt.it (E.C.);; 3 UOC Servizio di Radioterapia Oncologica, Dipartimento di Diagnostica per Immagini, Radioterapia Oncologica ed Ematologia, Fondazione Policlinico Universitario A. Gemelli IRCCS, Largo A. Gemelli 8, 00168 Rome, Italy; giuditta.chiloiro@policlinicogemelli.it (G.C.); angela.romano1@guest.policlinicogemelli.it (A.R.); elisa.meldolesi@policlinicogemelli.it (E.M.); flavia.degiacomo@guest.policlinicogemelli.it (F.D.G.); mariaantonietta.gambacorta@policlinicogemelli.it (M.A.G.); 4 UOS Medicina della Grande Obesità, Dipartimento di Scienze Mediche e Chirurgiche, Fondazione Policlinico Universitario A. Gemelli IRCCS, Largo A. Gemelli 8, 00168 Rome, Italy; 5 UOC Medicina Interna e Gastroenterologia, Dipartimento di Scienze Mediche e Chirurgiche, Fondazione Policlinico Universitario A. Gemelli IRCCS, Largo A. Gemelli 8, 00168 Rome, Italy; 6 Dipartimento di Scienze Radiologiche ed Ematologiche, Università Cattolica del Sacro Cuore, 00168 Rome, Italy

**Keywords:** EORTC QLQ-CAX24, rectal cancer, radiotherapy, cachexia, quality of life: body composition, muscle mass

## Abstract

Background: Patients with rectal cancer (RC) are at risk of developing cancer-related cachexia, a complex metabolic syndrome that can negatively impact quality of life (QoL), treatment tolerance, and clinical response. Objectives: The aim of the study was to explore the possible associations of the novel European Organization for Research and Treatment of Cancer QoL Questionnaire—Cancer Cachexia (EORTC QLQ-CAX24) scores with body composition parameters and physical performance in patients with locally advanced RC (LARC). Methods: This prospective observational study involved RC patients evaluated at the dedicated outpatient clinic of Clinical Nutrition at the Fondazione Policlinico Agostino Gemelli IRCCS. Patients with a confirmed diagnosis of LARC were enrolled between January and December 2023. The body composition parameters were measured using the preoperative computed tomography scan at the level of the third lumbar vertebra as well as using bioimpedance analysis before and after the radiotherapy treatment. QoL was measured by the EORTC QLQ-C30 and EORTC QLQ-CAX24 questionnaires. Results: A total of 56 RC patients were enrolled. Significant associations (*p* < 0.05) were found between EORTC QLQ-CAX24 values and the presence of cachexia, body composition, handgrip strength, and malnutrition diagnosis. Muscle mass was significatively also associated with EORTC QLQ-CAX24 results, suggesting a link between subjective perception of QoL and objectively measured body composition. Conclusions: The EORTC CAX24 questionnaire can be an effective tool for monitoring changes in cachexia status during radiotherapy, enabling early detection of cachexia-related complications and timely intervention.

## 1. Introduction

Rectal cancer (RC) has a complex epidemiological profile, with various risk factors contributing to its development and incidence. It is one of the most common types of gastrointestinal malignancies and represents the eighth most diagnosed cancer worldwide [1]. RC incidence and clinical characteristics have been extensively researched, showing an emerging trend of an increase in early rectal cancer and advanced adenomas, especially in the context of bowel screening programs [2]. Furthermore, the identification of unique clinical features in early-onset RC emphasizes the need for tailored management strategies [3]. RC risk factors include modifiable elements such as excessive body weight, lack of physical activity, tobacco use, alcohol consumption, and high red or processed meat intake, as well as non-modifiable factors like genetic predisposition, sex, and age [4]. Preventive measures targeting multiple lifestyle factors have shown promise in improving RC prevention. The joint effects of major lifestyle factors, including smoking, waist–hip ratio, alcohol consumption, diet, and exercise, have been investigated to understand their impact on RC risk. Adopting healthy lifestyles has been associated with a substantial reduction in cancer morbidity and mortality, underscoring the importance of prioritizing healthy behaviors for cancer prevention [5].

Locally advanced rectal cancer (LARC) is treated with neoadjuvant concurrent chemoradiotherapy, which is currently the preferred treatment approach for LARC to downstage the tumor before surgery [6]. The most common regimens for resectable RC include preoperative conventional chemoradiotherapy with delayed surgery and short-term radiotherapy with immediate surgery, which have been shown to reduce the local recurrence rate [7]. However, resistance to radiotherapy remains a significant hurdle in treating LARC [8,9,10].

Evaluations of body composition and muscle strength in cancer patients have had a growing interest in recent years. Different techniques can be used to detect muscle mass loss, both quantitative and qualitative loss, including bioelectrical impedance analysis (BIA), computed tomography (CT), and the handgrip strength test (HGS) [11].

BIA is a measurement technique that assesses the resistance of tissues to the passage of a small electric current to determine body composition. It is a guideline-accepted method for detecting muscle mass and it is widely used in the oncological setting. HGS is a test in which the force exerted by the hand and forearm is measured using a dynamometer, providing insight into muscle strength and physical function. CT is considered the gold-standard technique for non-invasive assessment of muscle mass, and it can be routinely utilized for assessing skeletal muscle mass without additional radiation, demonstrating its practicality, especially in oncological settings [12,13,14].

Indeed, CT-measured body composition has recently emerged as a valuable tool for assessing the prognosis of RC patients [15]. In patients with LARC undergoing neoadjuvant therapy, the volume of skeletal muscle mass appears to be linked to better prognosis and treatment tolerance [16]. The loss of muscle mass during neoadjuvant chemoradiotherapy is associated with worse survival outcomes in RC patients, enlightening the relevance of body composition indexes [17].

Cancer cachexia is a complex metabolic syndrome with physical and psychological symptoms, characterized by the progressive loss of body weight, muscle mass, and adipose tissue in individuals with cancer. This multifactorial condition is often associated with advanced stages of cancer and can be exacerbated by cancer treatments. Moreover, involuntary weight loss, which is the hallmark of cancer cachexia, influences therapy compliance and increases patients’ morbidity and mortality [18]. Additionally, cancer cachexia alone impairs quality of life (QoL), potentially leading to severe weight loss during chemotherapy, further emphasizing the multifaceted impact of this syndrome [19,20]. The presence of cancer cachexia is a significant concern due to its association with poor prognosis, decreased treatment tolerance, and negative impact on QoL, particularly in the physical, psychological, and social domains [21,22,23].

The EORTC QLQ-CAX24 is a specific questionnaire developed by the European Organization for Research and Treatment of Cancer (EORTC) to assess the impact of cancer cachexia on QoL. The questionnaire consists of 24 items that cover a wide range of physical, emotional, and social dimensions related to cancer patients’ experiences. These dimensions include pain, physical functioning, fatigue, appetite loss, sleep disturbances, body image, sexual functioning, and overall quality of life. The questionnaire allows patients to rate the severity of these symptoms and their impact on daily life using a specific scoring system. This specific questionnaire is employed in combination with the EORTC Core Quality of Life Questionnaire (QLQ-C30) to evaluate relevant symptoms and treatment side effects in cancer patients [24]. The EORTC QLQ-CAX24 has emerged recently as a valuable instrument for assessing the cachexia-specific quality of life in cancer patients, including those with advanced stages of the disease [25]. These questionnaires play a crucial role in enhancing patients’ well-being by enabling healthcare professionals to understand patients’ needs and provide appropriate support [26].

This prospective study aims to report the first data of QLQ-CAX24 on LARC patients and thus evaluate the relation with cachexia and other nutritional parameters.

## 2. Materials and Methods

### 2.1. Patients

Consecutive patients with a diagnosis of LARC (cT2–4, cN0–2, cM0), who were evaluated at the dedicated outpatient clinic of Clinical Nutrition at the Fondazione Policlinico Agostino Gemelli IRCCS in Rome between January and December 2023, were prospectively enrolled. Inclusion criteria were (a) patients with a confirmed diagnosis of rectal adenocarcinoma, (b) age over 18 years, and (c) patients confirmed to undergo neoadjuvant chemoradiotherapy by the institutional multidisciplinary tumor board. The study was conducted following the Declaration of Helsinki and approved by the Ethics Committee of CET Lazio Area 3 (protocol code ID 5022; 12 July 2022). Before initiating therapy (T0) and following the completion of radiotherapy (T1), all patients underwent a comprehensive nutritional and body composition assessment. This evaluation included detailed information on their dietary habits, daily caloric and protein intake, and the distribution of essential nutrients in their diets. These assessments aimed to identify any potential malnutrition issues they may have been experiencing. The Global Leadership Initiative on Malnutrition (GLIM) criteria were used to determine the presence and severity of malnutrition [27].

Cancer cachexia was defined as weight loss > 5% over the past 6 months (in the absence of simple starvation), BMI < 20 and any degree of weight loss > 2%, or appendicular skeletal muscle index consistent with sarcopenia (male < 7.26 kg/m^2^; female < 5.45 kg/m^2^) and any degree of weight loss > 2% [28]. All patients were followed by the clinical nutritionist and dietitian from the Clinical Nutrition Unit who prescribed a personalized diet combined with oral nutritional supplements (ONSs) or medical nutrition, if necessary, according to ESPEN Guidelines [29].

### 2.2. Body Composition Assessment

The body composition parameters were determined using CT simulation and analyzed at the level of the third lumbar vertebra, using dedicated software (SliceOmatic v5.0 from Tomovision), which analyzes various tissues based on their Hounsfield Unit (HU) analysis [30]. The following parameters were measured:

(a) Skeletal muscle area (SMA)—the total muscle area of the bilateral erector spinae, quadratus lumborum, psoas, internal and external obliques, transversus abdominis, and rectus abdominis;
(b) Inter-muscular adipose tissue (IMAT)—the adipose tissue within muscular fibers;
(c) Visceral adipose tissue (VAT)—the adipose tissue between internal organs;
(d) Subcutaneous adipose tissue (SAT)—the adipose tissue between the skin and muscular fascia;
(e) Muscle density (MD)—the mean Hounsfield Unit (HU) of SMA.

The following HU thresholds were used to quantify different parameters: −29 to +150 HU for SMA, −190 to −30 HU for SAT, −150 to −50 HU for VAT, and −190 to −30 HU for IMAT. The skeletal muscle index (SMI) was then calculated by normalizing SMA for squared height (in m^2^). According to the sex-specific definitions of Fearon et al., low muscle mass was defined as SMI < 52.4 cm^2^/m^2^ in men and SMI < 38.5 cm^2^/m^2^ in women [30]. For VAT analysis, the low-VAT group was defined as VAT < 160 cm^2^ for men and VAT < 80 cm^2^ for women [13,31].

Bioelectrical impedance analysis was conducted using BIA 101 (Akern^®^, Florence, Italy). The analysis includes values for parameters such as resistance, reactance, and phase angle, as well as derived data including fat-free mass (FFM), body cellular mass (BCM), body cellular mass index (BCMI), and total body water (TBW) [32]. The handgrip test assesses muscle strength by measuring the average of three consecutive measurements of maximum strength from the non-dominant hand [12]. Patients were defined as sarcopenic according to EWGSOP2 Criteria if patients had both low muscle strength and low muscle quantity or quality [12].

### 2.3. Quality of Life Questionnaires

Two questionnaires, the EORTC QLQ-C30 questionnaire and the EORTC QLQ-CAX24 questionnaire, were administered before and after radiotherapy to assess patients’ quality of life [24,25,26,33].

The QLQ-CAX24 questionnaire includes 24 items categorized into five multi-item symptom scales—food aversion (AV), eating and weight loss worry (EW), eating difficulties (EAT), loss of control (LC), and physical decline (PHY)—as well as three individual symptom scales for dry mouth (DM), indigestion or heartburn (IND), and forcing self to eat (FOR) and a single-item functional scale for adequate information on weight loss (INF) [24]. Following the manual scoring for each multi-item scale, the average points were calculated, obtaining the “raw scores”, and a linear transformation was applied to obtain a 0–100 value [34]. In functional scales, high values reflected a superior QoL, while in symptom scales, a high score was associated with a worse QoL.

### 2.4. Statistical Analysis

Statistical analysis was performed using STATA (version 18.0). The Shapiro–Wilk test was used to evaluate whether the continuous variables had a Gaussian distribution. Mean ± standard deviation was used to describe normally distributed variables, while median and interquartile range were used in other parameters. Student’s *t*-test and the Kruskal–Wallis test were used to compare continuous variables in the case of Gaussian and non-Gaussian distribution, respectively. Comparisons of proportions were performed with the Chi-Square or Fisher exact test, where appropriate. Type I error was set at 0.05 and statistical significance was defined when *p* < 0.05 (two-tailed).

## 3. Results

Fifty-six patients were enrolled according to the inclusion and exclusion criteria. Principal demographic and oncological data are reported in Table 1.

Patients were evenly distributed between genders, with a slight predominance of females (53.6%). Most patients were either normal weight or overweight, with a mean BMI of 25.6 ± 4.4 kg/m^2^. Risk of malnutrition according to NRS-2002 was detected in 27 patients (48.2%), while using GLIM criteria, 11 patients (21.4%) were deemed malnourished.

Regarding muscle strength assessment, the mean maximum hand grip strength (Fmax) was found to be 22.5 ± 9.6 kg; 44.6% of patients exhibited a reduced hand grip strength according to the pre-established cut-off.

Bioelectrical impedance analysis showed an average phase angle of 5.67 ± 0.98° and mean BCM of 26.9 ± 7.4 kg.

Concerning body composition parameters from CT scans, the mean SMI value was 41.5 ± 17.3 cm^2^/m^2^, and a low SMI was found in 13 patients (40.6%). The average MD was 33.5 ± 9.9 HU with reduced values in nine participants (28.1%). The average values of SMA, IMAT, and SAT were, respectively, 125.1 ± 35.9 cm^2^, 16.9 ± 8.9 cm^2^, and 260.9 ± 417.0 cm^2^. The medium VAT was 109.1 ± 61.2 cm^2^ and 16 participants (50.0%) were identified as having low VAT.

Combining data from muscle strength assessment and body composition, 12 patients (21.2%) were identified as sarcopenic.

Results regarding QoL are summarized in Table 2.

As for QLQ-C30, the highest mean scores were reported in cognitive functioning (89.5) and social functioning (86.2), whereas the lowest scores were observed in nausea and vomiting (2.38), financial difficulties (6.7), and dyspnea (8.6). Data from QLQ-CAX24 showed lower scores in eating difficulties (mean = 2.9, SD = 7.3), physical decline (mean = 5.9, SD = 11.2), and food aversion (mean = 6.7, SD = 10.7), while the highest scores were observed for dry mouth (mean = 17.7, SD = 27.9) and loss of control (mean = 19.2, SD = 20.3).

Table 3 summarizes the differences among QLQ-CAX24 tests according to cachexia.

Significant differences were found between cachectic patients (*n* = 31) and patients without cachexia (*n* = 25) in terms of several symptoms. Cachectic patients reported significantly higher levels of food aversion (9.9 vs. 0; *p* = 0.04), eating and weight loss worry (11.1 vs. 0; *p* = 0.02), and loss of control (16.7 vs. 11.1; *p* = 0.02). Additionally, dry mouth was significantly more prevalent in the cachectic group (33.3% vs. 0%; *p* = 0.01).

Data from clinical evaluation were compared with those derived from QLQ-CAX24 (Table 4).

Values are shown as medians and interquartile ranges (IQRs); *p* is the probability value; bold values represent statistically significant differences between groups.

Abbreviations: HGS, handgrip strength; GLIM, Global Leadership Initiative on Malnutrition criteria; MD, muscle density; NRS-2002, nutritional risk screening 2002; SMI, skeletal muscle index; VAT, visceral adipose tissue; BCM, Body Cell Mass; BCMI, Body Cell Mass Index.

Loss of control is significantly associated with sarcopenia (*p* = 0.02), low phase angle < 5.5 (*p* = 0.03), low muscle density < 28.6 HU (*p* = 0.04), and low scores on thehandgrip strength test (*p* = 0.001). Additionally, sarcopenic patients also exhibited a greater “food aversion” (*p* = 0.008) than the non-sarcopenic patients. Patients with low SMI were more likely to force themselves to eat compared to those with normal SMI (*p* = 0.03). Moreover, patients at risk of malnutrition according to NRS-2002 reported more frequent dry mouth compared to those not at risk (*p* = 0.03), and those malnourished according to GLIM criteria experienced greater food aversion and physical decline than non-malnourished patients.

**Figure 1:**
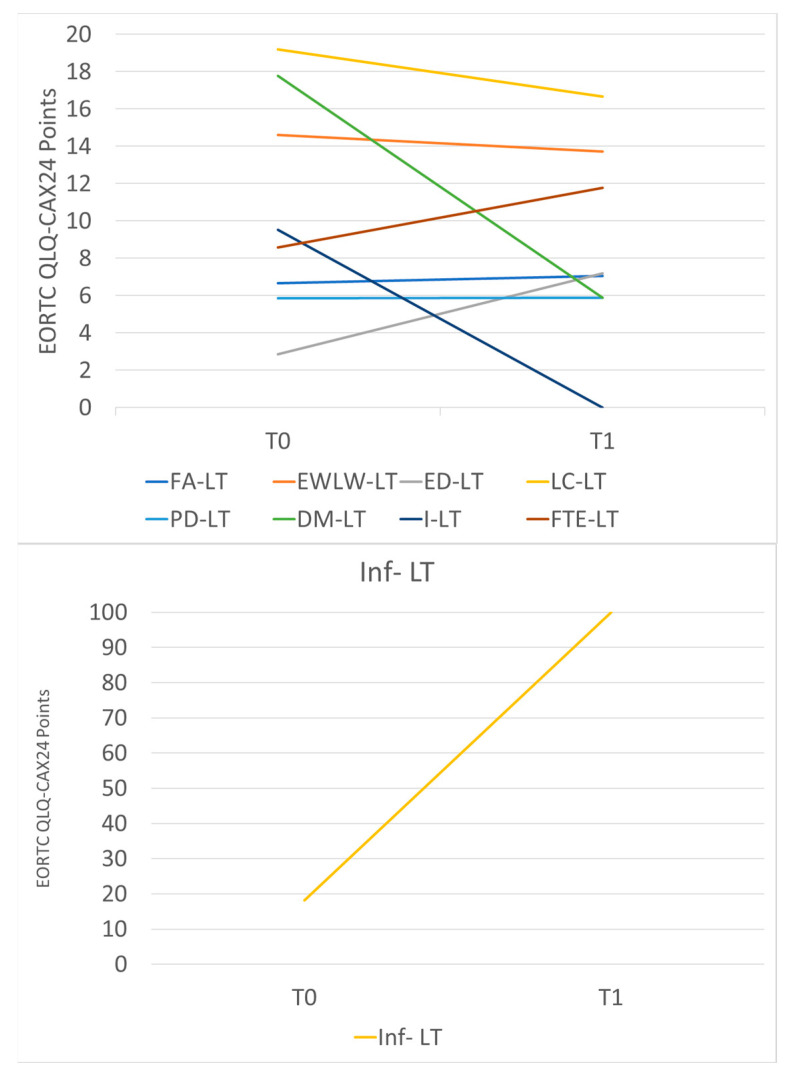
Variations in QLQ-CAX24 mean scores before and after the completion of radiotherapy. Abbreviations: FA-LT, food aversion; EWLW-LT, eating and weight loss worry; ED-LT, eating difficulties; LC-LT, loss of control; PD-LT, physical decline; DM-LT, dry mouth; I-LT, indigestion and Heartburn; FTE-LT, forcing self to eat; Inf-LT, adequate information about weight loss.

Table 5 and Figure 1 summarize the main differences between QLQ-CAX24 values before and after radiotherapy. In particular, there was a decrease in the dry mouth scale and an increase in adequate information about weight loss.

## 4. Discussion

To our knowledge, this paper represents the first comprehensive investigation of the QLQ-CAX24 questionnaire in patients diagnosed with RC. In patients with LARC, cancer-related cachexia significantly worsens outcomes, impacting treatment tolerance and survival rates [16]. The EORTC QLQ-CAX24 questionnaire could be a useful tool for assessing health-related QoL in this population [24]. However, since only a limited number of studies have evaluated its reliability in clinical settings, its practical application is not yet fully established.

The primary concern among these patients was the loss of control in their lives. This was closely followed by reports of dry mouth and significant worries related to eating and weight loss. A recent study by Luvián-Morales et al. validated the EORTC QLQ-CAX24 questionnaire specifically within a cohort of women suffering from cervical cancer [25]. Their findings were strikingly consistent with our results, as they also identified loss of control, concerns about eating and weight loss, and instances of dry mouth as the predominant issues faced by their patients. The alignment in concerns between these two distinct patient populations underscores the necessity for healthcare providers to address these psychological and physical challenges in their care strategies [35].

The associations between responses from the QLQ-CAX24 questionnaire and sarcopenia and malnutrition status were also explored, thereby validating its use in LARC patients. An analysis of body weight data from the initial nutritional evaluation revealed that, at that time, the average BMI of the patients exceeded 25 kg/m^2^, indicating an overweight condition. However, clinical evaluations showed that a significant percentage of patients were either at risk of malnutrition (48.2%) or already malnourished according to GLIM criteria (21.4%). Additionally, the prevalence of sarcopenia among the patients was also found to be 21.4%. Significant results have been demonstrated between scores from specific areas assessed by the QLQ-CAX24 test and the measures of sarcopenia and malnutrition detected using validated clinical instruments. Notably, “loss of control” was significantly associated with low physical activity, reduced muscle density, and decreased muscle strength. Lower SMI values measured from CT scan analysis were linked to higher scores on the “forcing self to eat” item, and sarcopenia status was associated with food aversion. Furthermore, food aversion and physical decline were more prevalent in malnourished patients according to the GLIM criteria compared with non-malnourished patients.

Patients at risk of malnutrition according to NRS-2002 reported experiencing a “dry mouth”. Thus, the QLQ-CAX24 questionnaire appears to be a valuable tool for identifying patients at risk of cachexia and for monitoring the impact of this condition on their quality of life.

By using the QLQ-CAX24 questionnaire in the context of RC patients undergoing radiotherapy, clinicians could gain a comprehensive assessment of the presence and impact of cachexia in these individuals. This information could guide treatment decisions, supportive care interventions, and surveillance strategies to address the specific needs of patients experiencing cachexia during their radiotherapy treatment. Moreover, an increase in awareness and information about weight loss, alongside a decrease in instances of dry mouth, should also be considered.

This study has some limitations. The most important ones are the single-center nature of the study and the small sample size, which can impact the reliability of findings that need to be confirmed. Moreover, the QoL and body composition assessment should be monitored a few weeks after radiotherapy completion since the effects of radiotherapy could be late-onset and long-lasting; thereby, the nutritional follow-up of patients is necessary even after the termination of the oncologic course. To gain a deeper understanding of how cachexia affects treatment outcomes in RC patients undergoing radiotherapy, larger prospective studies should be conducted. These studies ought to involve patients with other types of cancer including comprehensive assessments of cachexia, using multiple nutritional evaluation tools in addition to the QLQ-CAX24 questionnaire. Furthermore, there is increasing interest in the potential role of exercise in enhancing physical performance, body composition, and QoL during neoadjuvant chemoradiotherapy. This suggests that a multifaceted approach is necessary to effectively address the impact of body composition on patient outcomes [36].

## 5. Conclusions

The EORTC QLQ-CAX24 questionnaire offers valuable insights into the prevalence and severity of cachexia, as well as its impact on treatment outcomes and QoL. This questionnaire is a valuable tool for tracking changes in the cachexia status of LARC patients during radiotherapy. It is closely related to the diagnosis of cachexia and may allow for more timely intervention. While the current study highlights the relationship between the EORTC QLQ-CAX24 questionnaire, cachexia, and QoL in RC patients undergoing radiotherapy, there is a clear need for further research to build upon its findings.

## Figures and Tables

**Table 1:** Baseline characteristics of patients including body composition measurements.

Variables	*n* = 56
Female sex, *n* (%)	30 (53.6%)
Weight (kg), mean ± SD	68.2 ± 12.9
Height (cm), mean ± SD	163.6 ± 11.1
BMI (kg/m^2^), mean ± SD	25.6 ± 4.4
At risk of malnutrition, *n* (%)	27 (48.2%)
Malnourished patients ^#^, *n* (%)	11 (25.6%)
Handgrip F_max_ (kg), mean ± SD	22.5 ± 9.6
Low HGS *, *n* (%)	25 (44.6%)
Sarcopenic patients, *n* (%)	12 (21.4%)
Cachexia, *n* (%)	31 (55.4%)
BIA body composition parameters	*n* = 56
Resistance (W), mean ± SD	516.6 ± 78.2
Reactance (W), mean ± SD	50.7 ± 8.2
Phase Angle (°), mean ± SD	5.67 ± 0.98
Total Body Water (L), mean ± SD	55.0 ± 6.8
Extra-Cellular Water (L), mean ± SD	47.7 ± 4.8
Fat Mass (kg), mean ± SD	16.9 ± 8.0
Fat Mass (%), mean ± SD	24.4 ± 9.5
Fat-Free Mass (kg), mean ± SD	51.1 ± 9.8
Fat-Free Mass (%), mean ± SD	75.3 ± 9.7
Body Cell Mass (kg), mean ± SD	26.9 ± 7.4
Body Cell Mass Index (kg/m^2^), mean ± SD	9.9 ± 1.8
Basal Metabolic Rate (kcal), mean ± SD	1532 ± 214
CT scan body composition parameters	*n* = 32
Skeletal Muscle Area (cm^2^), mean ± SD	125.1 ± 35.9
Skeletal Muscle Index (cm^2^/m^2^), mean ± SD	41.5 ± 17.3
Low SMI ^§^, *n* (%)	13 (40.6%)
Muscle Density (HU), mean ± SD	33.5 ± 9.9
Low Muscle Density ^@^, *n* (%)	9 (28.1%)
Inter-Muscular Adipose Tissue (cm^2^), mean ± SD	16.9 ± 8.9
VAT (cm^2^), mean ± SD	109.1 ± 61.2
Low VAT ^^^, *n* (%)	16 (50.0%)
Subcutaneous Adipose Tissue (cm^2^), mean ± SD	260.9 ± 417.0

^#^ According to GLIM Criteria. * Low HGS was defined as HGS < 27 kg for men and HGS < 16 kg for women. ^§^ SMI < 52.4 cm^2^/m^2^ in men and SMI < 38.5 cm^2^/m^2^ in women. ^@^ Muscle density < 28.6 HU. ^^^ VAT < 160 cm^2^ for men and VAT < 80 cm^2^ for women. Abbreviations: BIA: bioimpedance analysis; BMI: body mass index; HGS: handgrip strength; HU: Hounsfield Unit; SD: standard deviation; SMI: skeletal muscle index; VAT: visceral adipose tissue.

**Table 2:** Quality of life values according to QLQ-C30 and QLQ-CAX24.

	Mean (SD)	Median (IQR)	Minimum (%)	Maximum (%)
**QLQ C30**				
Global health	65.6 (21.5)	66.7 (50–83.3)	25	100
*Functional Scales*				
Physical	82.7 (18.7)	86.7 (73.3–100)	40	100
Role	81.0 (26.5)	100 (66.7–100)	16.7	100
Emotional	79.6 (21.2)	83.3 (66.7–100)	33.3	100
Cognitive	89.5 (18.1)	100 (83.3–100)	33.3	100
Social	86.2 (17.8)	100 (66.7–100)	50	100
*Symptom Scales*				
Fatigue	26.7 (26.4)	33.3 (0–33.3)	0	100
Nausea and Vomiting	2.38 (7.1)	0 (0–0)	0	33.3
Pain	23.8 (27.8)	16.7 (0–50)	0	83.3
Dyspnea	8.6 (14.8)	0 (0–33.3)	0	33.3
Insomnia	23.8 (31.9)	0 (0–33.3)	0	100
Appetite loss	13.3 (23.2)	0 (0–33.3)	0	66.7
Constipation	20.9 (29.2)	0 (0–33.3)	0	100
Diarrhea	22.8 (33.1)	0 (0–33.3)	0	100
Financial Difficulties	6.7 (17.7)	0 (0–0)	0	66.7
**QLQ-CAX24**				
Food aversion	6.7 (10.7)	0 (0–13.3)	0	100
Eating and weight loss worry	14.6 (19.4)	0 (0–22.2)	0	66.7
Eating difficulties	2.9 (7.3)	0 (0–0)	0	66.7
Loss of control	19.2 (20.3)	16.7 (0–27.8)	0	83.3
Physical decline	5.9 (11.2)	0 (0–11.1)	0	33.3
Dry mouth	17.7 (27.9)	0 (0–33.3)	0	100
Indigestion and Heartburn	9.5 (19.1)	0 (0–0)	0	66.7
Forcing self to eat	8.6 (21.9)	0 (0–0)	0	100
Adequate information about weight loss	12.8 (19.7)	0 (0–33.3)	0	66.7

Abbreviations: IQR, interquartile range; QLQ C30, Quality of Life Questionnaire—Cancer; QLQ-CAX24, Quality of Life Questionnaire—Cancer Cachexia; SD, standard deviation.

**Table 3:** QLQ-CAX24 quality of life values according to cachexia diagnosis.

	Cachexia (31 Patients)	No Cachexia (25 Patients)	*p*
Food aversion	9.9 (0–20.3)	0 (0–6.7)	0.04 *
Eating and weight loss worry	11.1 (0–33.3)	0 (0–22.2)	0.02 *
Eating difficulties	0 (0–0)	0 (0–0)	0.67
Loss of control	16.7 (5.6–33.3)	11.1 (0–27.8)	0.02 *
Physical decline	0 (0–22.2)	0 (0–0)	0.57
Dry mouth	33.3 (0–66.7)	0 (0–0)	0.001 *
Indigestion and Heartburn	0 (0–0)	0 (0–0)	0.18
Forcing self to eat	0 (0–0)	0 (0–0)	0.63
Adequate information about weight loss	0 (0–0)	0 (0–33.3)	0.84

* Significant associations (*p* < 0.05).

**Table 4:** Associations of QLQ-CAX24 values with nutritional parameters.

Phase Angle	Phase Angle < 5.5 28 Patients	Phase Angle ≥ 5.5 28 Patients	*p*
Food aversion	0 (0–13.3)	0 (0–6.7)	0.24
Eating and weight loss worry	11.1 (0–33.3)	0 (0–11.1)	0.18
Eating difficulties	0 (0–11.1)	0 (0–0)	0.07
Loss of control	22.2 (11.1–33.3)	5.5 (0–27.8)	0.03
Physical decline	0 (0–11.1)	0 (0–0)	0.41
Dry mouth	0 (0–33.3)	0 (0–33.3)	0.27
Indigestion and Heartburn	0 (0–33.3)	0 (0–0)	0.34
Forcing self to eat	0 (0–0)	0 (0–0)	0.45
Adequate information about weight loss	0 (0–11.1)	0 (0–0)	0.24
**BCM**	**BCMI < 9.8 kg/m^2^** **30 patients**	**BCMI ≥ 9.8 kg/m^2^** **26 patients**	***p***
Food aversion	6.7 (0–20)	0 (0–6.6)	0.11
Eating and weight loss worry	0 (0–33.3)	5.5 (0–22.2)	0.94
Eating difficulties	0 (0–0)	0 (0–0)	0.57
Loss of control	22.2 (0–33.3)	11.1 (0–27.7)	0.19
Physical decline	0 (0–11.1)	0 (0–0)	0.36
Dry mouth	0 (0–33.3)	0 (0–33.3)	0.90
Indigestion and Heartburn	0 (0–0)	0 (0–16.6)	0.93
Forcing self to eat	0 (0–0)	0 (0–0)	0.51
Adequate information about weight loss	0 (0–0)	0 (0–0)	0.17
CT scan-derived Body Composition Parameters			
**SMI**	**Low SMI** ^**§**^ **13 patients**	**Normal SMI** ^**§**^ **19 patients**	***p***
Food aversion	0 (0–20)	0 (0–6.7)	0.42
Eating and weight loss worry	11.1 (0–33.3)	0 (0–22.2)	0.22
Eating difficulties	0 (0–0)	0 (0–0)	0.83
Loss of control	16.7 (5.5–27.7)	16.7 (5.5–33.3)	0.95
Physical decline	0 (0–22.2)	0 (0–11.1)	0.89
Dry mouth	0 (0–33.3)	0 (0–0)	0.20
Indigestion and Heartburn	0 (0–33.3)	0 (0–33.3)	0.94
Forcing self to eat	11.1 (0–33.3)	0 (0–0)	0.03
Adequate information about weight loss	0 (0–0)	0 (0–16.7)	0.85
**MD**	**Low MD < 28.6 HU** **13 patients**	**Normal MD > 28.6 HU** **19 patients**	***p***
Food aversion	0 (0–13.3)	0 (0–13.3)	0.89
Eating and weight loss worry	11.1 (0–22.2)	0 (0–22.2)	0.65
Eating difficulties	0 (0–0)	0 (0–0)	0.79
Loss of control	22.2 (16.6–33.3)	11.1 (0–27.7)	0.04
Physical decline	0 (0–11.1)	0 (0–11.1)	0.97
Dry mouth	16.6 (0–61.1)	0 (0–0)	0.11
Indigestion and Heartburn	0 (0–33.3)	0 (0–0)	0.47
Forcing self to eat	0 (0–0)	0 (0–0)	0.22
Adequate information about weight loss	0 (0–0)	0 (0–0)	0.13
**VAT**	**Low VAT ^** **13 patients**	**Normal VAT ^** **19 patients**	***p***
Food aversion	0 (0–13.3)	0 (0–13.3)	0.72
Eating and weight loss worry	11.1 (0–22.2)	0 (0–33.3)	0.93
Eating difficulties	0 (0–0)	0 (0–0)	0.90
Loss of control	16.6 (5.5–27.7)	16.6 (11.1–33.3)	0.24
Physical decline	0 (0–0)	0 (0–22.2)	0.22
Dry mouth	0 (0–0)	0 (0–55.5)	0.07
Indigestion and Heartburn	0 (0–0)	0 (0–33.3)	0.17
Forcing self to eat	0 (0–0)	0 (0–0)	0.54
Adequate information about weight loss	0 (0–0)	0 (0–16.6)	0.85
**Others**			
**Handgrip Strength Test**	Low HGS * **25 patients**	Normal HGS * **31 patients**	***p***
Food aversion	0 (0–13.3)	0 (0–9.9)	0.67
Eating and weight loss worry	0 (0–22.2)	11.1 (0–27.8)	0.53
Eating difficulties	0 (0–0)	0 (0–11.1)	0.64
Loss of control	22.2 (11.1–33.3)	5.5 (0–22.2)	0.02
Physical decline	0 (0–22.2)	0 (0–0)	0.27
Dry mouth	0 (0–55.5)	0 (0–33.3)	0.79
Indigestion and Heartburn	0 (0–0)	0 (0.16.7)	0.70
Forcing self to eat	0 (0–0)	0 (0–0)	0.51
Adequate information about weight loss	0 (0–33.3)	0 (0–33.3)	0.94
**Sarcopenic Patients**	**Not Sarcopenic** **44 patients**	**Sarcopenic** **12 patients**	***p***
Food aversion	0 (0–6.7)	20 (0–26.7)	0.008
Eating and weight loss worry	0 (0–22.2)	11.1 (0–44.4)	0.20
Eating difficulties	0 (0–0)	0 (0–11.1)	0.06
Loss of control	5.6 (0–22.2)	33.3 (16.7–33.3)	0.001
Physical decline	0 (0–0)	0 (0–22.2)	0.27
Dry mouth	0 (0–16.7)	33.3 (0–55.6)	0.13
Indigestion and Heartburn	0 (0–0)	0 (0–33.3)	0.23
Forcing self to eat	0 (0–0)	0 (0–0)	1.0
Adequate information about weight loss	0 (0–33.3)	0 (0–0)	0.51
**At risk of Malnutrition**	**NRS-2002 < 3** **29 patients**	**NRS-2002 ≥ 3** **27 patients**	***p***
Food aversion	0 (0–6.6)	6.6 (0–20)	0.10
Eating and weight loss worry	0 (0–22.2)	11.1 (0–27.7)	0.34
Eating difficulties	0 (0–0)	0 (0–0)	0.83
Loss of control	11.1 (0–27.7)	16.6 (2.7–30.6)	0.58
Physical decline	0 (0–0)	0 (0–25)	0.07
Dry mouth	0 (0–0)	33.3 (0–50)	0.03
Indigestion and Heartburn	0 (0–0)	0 (0–16.6)	0.88
Forcing self to eat	0 (0–0)	0 (0–16.6)	0.42
Adequate information about weight loss	0 (0–33.3)	0 (0–0)	0.69
**Malnourished Patients**	**GLIM-negative** **32 patients**	**GLIM-positive** **11 patients**	***p***
Food aversion	0 (0–6.6)	13.3 (0–20)	0.02
Eating and weight loss worry	0 (0–22.2)	16.6 (0–33.3)	0.22
Eating difficulties	0 (0–0)	0 (0–0)	0.95
Loss of control	13.9 (0–27.7)	22.2 (0–33.3)	0.56
Physical decline	0 (0–0)	11.1 (0–27.7)	0.02
Dry mouth	0 (0–16.6)	33.3 (0–33.3)	0.11
Indigestion and Heartburn	0 (0–16.6)	0 (0–0)	0.72
Forcing self to eat	0 (0–0)	0 (0–33.3)	0.26
Adequate information about weight loss	0 (0–33.3)	0 (0–0)	0.09

* Low HGS was defined as HGS < 27 kg for men and HGS < 16 kg for women. ^§^ SMI < 52.4 cm^2^/m^2^ in men and SMI < 38.5 cm^2^/m^2^ in women. ^ VAT < 160 cm^2^ for men and VAT < 80 cm^2^ for women.

**Table 5:** Variations in QLQ-CAX24 values before and after the completion of radiotherapy.

QLQ-CAX24	Before RT	After RT	*p*
Food aversion	6.7 (10.7)	7.0 (7.9)	0.82
Eating and weight loss worry	14.6 (19.4)	13.7 (23.7)	0.23
Eating difficulties	2.9 (7.3)	7.2 (10.3)	0.49
Loss of control	19.2 (20.3)	16.7 (17.5)	0.66
Physical decline	5.9 (11.2)	5.8 (7.9)	0.62
Dry mouth	17.7 (27.9)	5.8 (13.0)	0.04 *
Indigestion and Heartburn	9.5 (19.1)	0 (0)	0.08
Forcing self to eat	8.6 (21.9)	11.7 (16.4)	0.67
Adequate information about weight loss	12.8 (19.7)	90 (12.4)	0.01 *

* Significant results (*p* < 0.05); abbreviations: RT, radiotherapy.

## Data Availability

The data presented in this study are available on request from the corresponding author. The data are not publicly available due to privacy and are part of an ongoing study.
